# Growth of Nanotubular Flowers Using Environment-Friendly Electrolytes and their Biomedical Applications

**DOI:** 10.1021/acsomega.6c00587

**Published:** 2026-08-10

**Authors:** Niharika Rawat, Metka Benčina, Veronika Kralj Iglič, Katja Lakota, Polona Žigon, Barbara Šetina-Batič, Aleš Iglič, Janez Kovač, Ita Junkar

**Affiliations:** † Laboratory of Physics, Faculty of Electrical Engineering, 68934 University of Ljubljana, Tržaška 25 1000, Ljubljana, Slovenia; ‡ 61790 Jožef Stefan Institute, Jamova cesta 39 1000, Ljubljana, Slovenia; § Laboratory of Clinical Biophysics, Faculty of Health Sciences, University of Ljubljana, Zdravstvena pot 5 1000, Ljubljana, Slovenia; ∥ Department of Rheumatology, 37667 University Medical Centre Ljubljana, Vodnikova 62 1000, Ljubljana, Slovenia; ⊥ Institute of Metals and Technology, Lepi pot 11 1000, Ljubljana, Slovenia

## Abstract

Plasma-induced electrochemical anodization (PA) is introduced as an alternative strategy for fabricating titanium oxide nanostructures using environmentally benign, chloride-based electrolytes. Unlike conventional anodization methods that rely on metallic cathodes and fluoride-containing acidic electrolytes, the PA approach employs an atmospheric-pressure plasma jet (APPJ) as the cathode, enabling anodization in aqueous sodium chloride solutions with optional ethylene glycol addition. By adjusting electrolyte composition and processing conditions, hierarchical flower-like microstructures composed of densely packed nanotube-like TiO_2_ features were obtained. The most uniform and well-defined structures were obtained with 2.5 M NaCl and ethylene glycol. Additional oxygen plasma treatment modified surface chemistry and enhanced wettability without altering surface morphology. Biological evaluation demonstrated that PA-treated surfaces significantly reduced platelet adhesion and activation, decreased *Escherichia coli* attachment by up to 92%, and promoted endothelial cell adhesion and spreading. Smooth muscle cells also exhibited increased adhesion to modified surfaces; however, their morphology was altered, with reduced spreading and a nonuniform distribution. Overall, the more pronounced response of endothelial cells indicates a favorable trend toward endothelialisation. These findings demonstrate that plasma-induced electrochemical anodization enables the fabrication of titanium surfaces with multifunctional biological performance, combining reduced thrombogenicity and bacterial adhesion with enhanced endothelial response. The use of fluoride-free electrolytes further highlights the potential of this approach for applications in cardiovascular and other blood-contacting biomedical devices.

## Graphical abstract

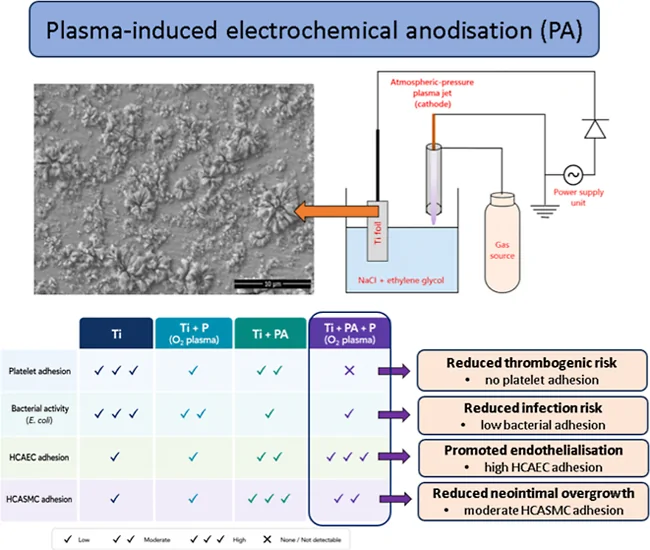

## 1 Introduction

Titanium dioxide (TiO_2_) has attracted sustained interest owing to its favorable electrical and optical properties, chemical stability, photocatalytic activity, and excellent biocompatibility.^1,2^ In biomedical applications, nanostructured TiO_2_ surfaces are particularly relevant because they can modulate protein adsorption, cellular responses, and interfacial biological events, making them attractive for use in implants and blood-contacting devices. Among various fabrication approaches, titanium dioxide nanotubular surfaces (NTs) have been extensively studied due to their high surface area and tunable geometry. NTs have been fabricated using methods such as hydrothermal synthesis,^3^ sol–gel processing,^3^ electrochemical anodization,^4,5^ and chemical vapor deposition.^6^ Of these, electrochemical anodization remains the most widely used technique because it is cost-effective and allows precise control over nanotube dimensions through parameters such as voltage, electrolyte composition, temperature, and anodization time.^7–9^ In conventional anodization, nanotube formation relies on a dynamic equilibrium between anodic oxide growth and chemical dissolution, typically mediated by fluoride ions.^8–11^ This process generally requires aggressive and hazardous electrolytes, including hydrofluoric acid, sulfuric acid, or perchloric acid, which raise concerns regarding operator safety, environmental impact, and industrial scalability.^7,12^ As a result, considerable effort has been directed toward developing alternative electrolyte systems that eliminate fluoride while still enabling controlled nanostructure formation. Dilute nitric acid has been used to generate nanoporous or nanotubular TiO_2_, in which nitrate ions form unstable complexes that decompose during anodization, yielding TiO_2_.^13,14^ Other approaches include electrolytes containing hydrogen peroxide, or mixed oxidizing systems that accelerate oxide growth by generating reactive oxygen species.^15,25,26^ Chloride-containing electrolytes have also been investigated, as chloride ions can induce localized passivity breakdown and pitting corrosion on titanium, leading to the formation of high-aspect-ratio nanotubular bundles under specific anodization conditions.^11,16,17,43,44^ Replacing fluoride ions with chloride therefore, represents a less toxic and potentially more sustainable route to titanium nanostructuring.

In parallel with electrolyte engineering, plasma-assisted electrochemical approaches have emerged as environmentally friendly alternatives for nanomaterial synthesis.^18^ In plasma electrochemistry, reactive species generated at the plasma–liquid interface drive electrochemical reactions without the need for toxic reducing or stabilizing agents.^19–21^ Early studies demonstrated the synthesis of TiO_2_ nanoparticles using pulsed plasma discharges in water,^22^ while subsequent work employed plasma-treated water as an anodizing electrolyte, exploiting plasma-generated hydrogen peroxide and nitrate species to promote oxide growth.^23,24^ It has been shown that such plasma-activated species can independently induce porous or nanotubular TiO_2_ formation during anodization.^13,14^ More recently, hybrid plasma–anodization strategies have been proposed, where plasma-treated water is continuously supplied during anodization to maintain a steady flux of reactive species, enabling rapid formation of TiO_2_ nanotubes.^27^

Atmospheric-pressure plasma jets (APPJs) are particularly versatile plasma sources for nanofabrication and biomedical applications because they operate in ambient conditions, are easily integrated into electrochemical systems, and generate a rich mixture of reactive oxygen and nitrogen species.^28,31^ Fang et al. demonstrated that an APPJ can function as a counter electrode in liquid-phase electrochemical processes, enabling plasma-assisted formation of oxide layers and nanoparticles directly on submerged electrodes.^32^ Similar concepts have been applied to fabricate porous electrocatalysts and functional coatings using rapid, plasma-driven processes.^33^ In addition to plasma-assisted anodization, low-pressure oxygen plasma treatment is widely used as a postprocessing step to tailor surface chemistry, enhance wettability, and remove adventitious carbon contamination without significantly altering surface topography.^29,30,34–37^

In this work, we present a plasma-induced electrochemical anodization (PA) strategy for the fabrication of titanium oxide nanostructures using environmentally benign, chloride-based electrolytes. In this approach, the conventional solid cathode is replaced by an atmospheric-pressure plasma jet, which serves as the cathode during anodization, while titanium acts as the anode. By tuning electrolyte composition, anodization time, and plasma parameters, we demonstrate the formation of hierarchical flower-like microstructures composed of densely packed TiO_2_ nanotube bundles. An additional low-pressure oxygen plasma treatment is employed to modify surface chemistry and wettability without altering the surface morphology. The biological performance of the resulting surfaces is evaluated for hemocompatibility, antibacterial activity, and cellular response using human coronary artery endothelial cells (HCEC) and human coronary artery smooth muscle cells (HCASMC). To our knowledge, this study represents the first systematic investigation of the biological response to nanostructures fabricated via plasma-induced electrochemical anodization, highlighting its potential as a sustainable and versatile platform for engineering titanium surfaces for cardiovascular and other blood-contacting biomedical applications.

While fluoride-free anodization and plasma-assisted approaches have been previously reported, their combination with comprehensive biological evaluation remains limited, with most studies focusing either on the synthesis of TiO_2_ nanostructures using alternative electrolytes or on isolated biological end points, such as antibacterial activity or cell response. In contrast, the present study introduces several key advances by integrating (i) a plasma-induced electrochemical anodization configuration employing an atmospheric-pressure plasma jet as the cathode, enabling anodization in simple chloride-based electrolytes without the need for conventional metallic counter-electrodes or hazardous fluoride-containing chemistries, (ii) the formation of a distinct hierarchical “flower-like” TiO_2_ nanotubular morphology arising from a chloride-induced localized breakdown mechanism, which differs fundamentally from the ordered arrays typically obtained in conventional anodization systems, and (iii) a systematic and integrated biological evaluation performed on identical surfaces, including platelet adhesion, bacterial attachment (*E. coli*), endothelial cell (HCEC) behavior, and smooth muscle cell (HCASMC) response. This combined approach enables direct correlation between processing conditions, surface morphology, wettability, and multiple biologically relevant end points. To the best of our knowledge, this is the first study to integrate these aspects into a unified framework for designing multifunctional TiO_2_ surfaces using environmentally benign processing routes.

## 2 Experimental Section

### 2.1 Materials

Titanium (Ti) foils (Advent, thickness 0.10 mm, purity ≥ 99.6%) were used as substrates for all experiments. Prior to anodization, the foils were sequentially ultrasonically cleaned in acetone, ethanol, and deionized water for 5 min each, then dried under a nitrogen stream. Deionized water (Milli-Q, Merck KGaA, Darmstadt, Germany), phosphate-buffered saline (PBS; Sigma-Aldrich, St. Louis, MO, USA), sodium chloride (NaCl, ≥ 99.5%, Fluka), and ethylene glycol (EG, ≥ 99.5%, Fluka) were used as electrolytes without further purification.

### 2.2 Plasma-Induced Electrochemical Anodisation (PA)

Plasma-induced electrochemical anodization (PA) was performed at room temperature using a custom-built atmospheric-pressure plasma jet (APPJ) as the cathode and titanium foil as the anode, as schematically illustrated in Figure 1. A constant voltage of 60 V was applied for anodization times of 30 or 60 min. The titanium foil was positioned vertically opposite the plasma jet at a distance of 1–2 cm and fully immersed in the electrolyte. The APPJ was operated using a radio frequency (RF) power supply at 375 kHz and consisted of a tubular dielectric capillary (glass or quartz) with an internal powered electrode and an external grounded ring electrode located near the gas outlet. Argon was used as the working gas, with flow rates controlled by mass flow controllers (up to 1000 sccm). PA experiments were conducted in four environmentally benign electrolytes: deionized water, PBS, and aqueous NaCl solutions at 0.25 and 2.5 M. Unless otherwise stated, anodization was performed for 60 min. To investigate the influence of anodization parameters on surface nanostructuring, selected samples were treated for 30 or 60 min using NaCl electrolytes of different concentrations (0.25 and 2.5 M), as shown in Figure 3. The surface morphology of the anodized titanium foils was analyzed by scanning electron microscopy (SEM).

**Figure 1:**
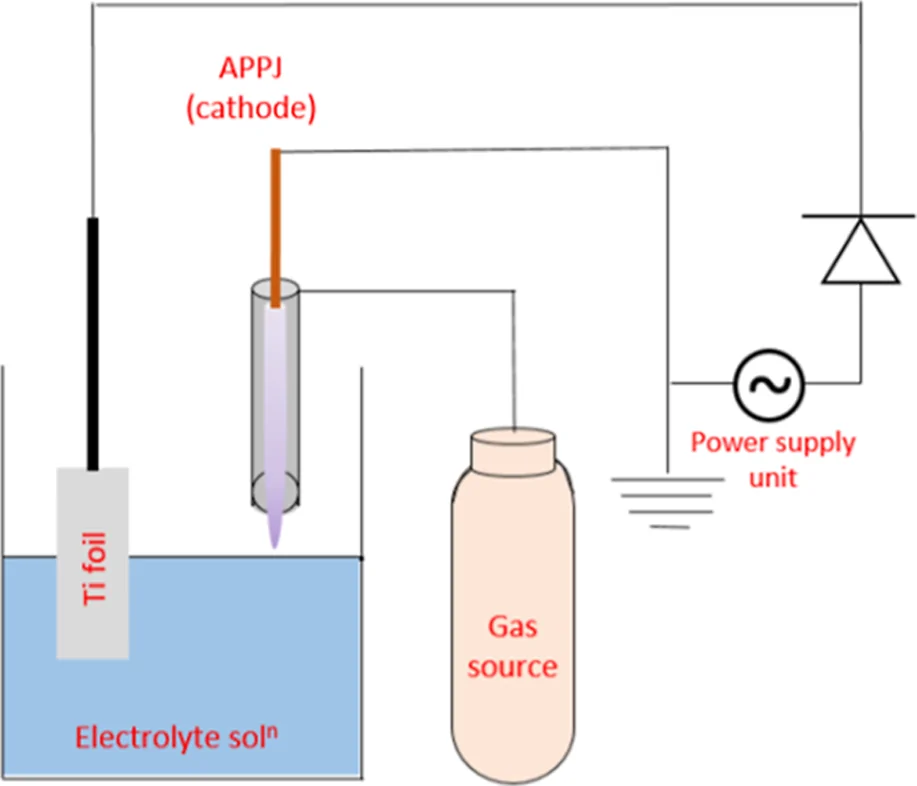
Schematic of a setup used for Atmospheric pressure plasma jet-induced electrochemical anodization.

**Figure 2:**
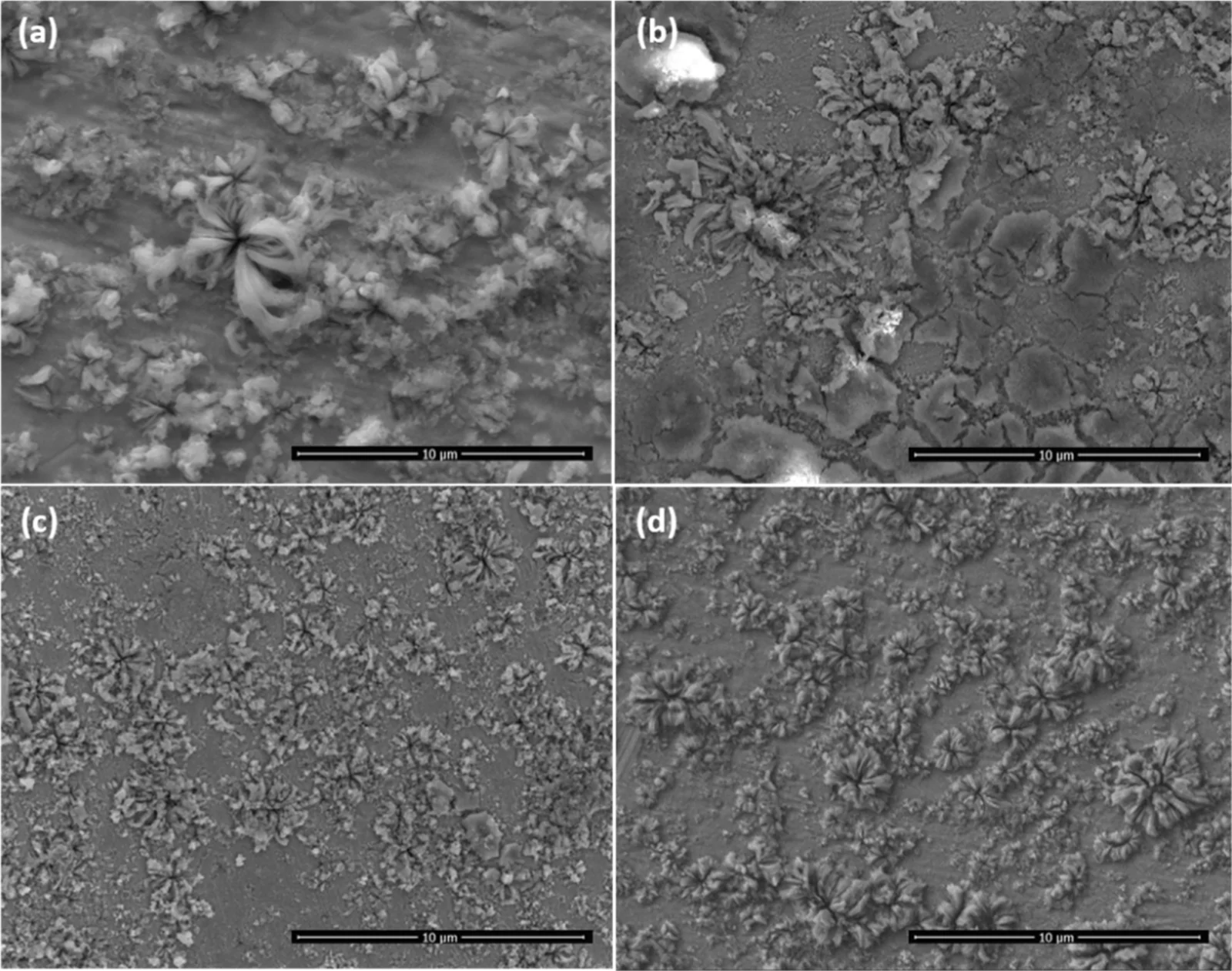
Scanning electron morphology (SEM) images of titanium foil subjected to plasma anodization in different electrolytes (a) Deionized water (b) PBS (c) 2.5 M sodium chloride (NaCl) and (d) 2.5 M sodium chloride (NaCl) + Ethylene Glycol (EG) for 1 h.

**Figure 3:**
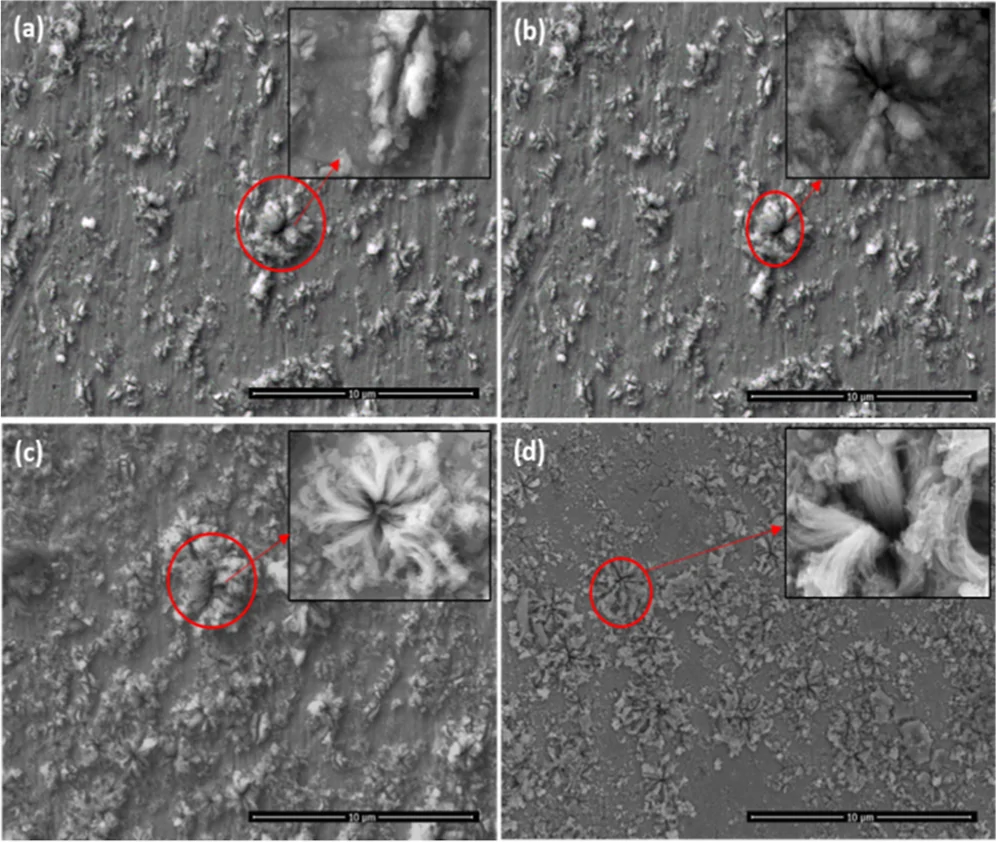
SEM images of Ti foil subjected to plasma anodization in electrolyte solution containing (a) 0.25 M NaCl for 30 min, (b) 0.25 M NaCl for 60 min, (c) 2.5 M NaCl for 30 min and (d) 2.5 M NaCl for 60 min (Insets 1 μm).

### 2.3 Oxygen Plasma Treatment

Fabricated nanostructures were treated with low-pressure oxygen plasma in a plasma reactor.^38,39^ The plasma was generated using an inductively coupled radio frequency (RF) source operating at 13.56 MHz with an output power of approximately 200 W. High-purity oxygen was introduced into the discharge chamber, and the operating pressure was maintained at 75 Pa, corresponding to the highest degree of molecular dissociation as determined by catalytic probe measurements. Samples were placed on a glass holder and exposed to oxygen plasma for 60 s.

### 2.4 Hemocompatibility Studies

The adhesion and activation of platelets on the samples were performed in accordance with the Declaration of Helsinki and approved by Slovenia’s ethics committee (approval number 56/03/10). Before whole-blood incubation, samples were cleaned with ethanol, dried, and incubated with blood obtained by venipuncture from a healthy human donor. The blood was drawn into trisodium citrate anticoagulant-coated 9 mL tubes. Afterward, the fresh blood was incubated with samples in 24-well plates for 45 min at room temperature. After incubation, PBS was added to the samples, which was later removed, and the sample surface was rinsed 5 times with 250 μL PBS to remove weakly adherent platelets. Adherent cells were fixed with 250 μL of 0.5% glutaraldehyde (GA) solution for 15 min at room temperature. Afterward, the surfaces were rinsed with PBS and then dehydrated using a graded ethanol series (50, 70, 80, 90, 100, and again 100 vol % ethanol) for 5 min, and in the final stage of the series (100 vol % ethanol) for 15 min. Then the samples were placed in a critical-point dryer, where the solvent was exchanged with liquid carbon dioxide. By increasing the temperature in the drier, the liquid carbon dioxide passes through the critical point, at which its density equals that of the vapor phase. This drying process preserves the sample’s natural structure and avoids surface tension that can occur during normal drying. The dried samples were coated with a gold/palladium layer and examined using scanning electron microscopy (SEM). The experiment was performed in triplicate. For quantitative analysis, SEM images were acquired at identical magnification, and nine independent fields per sample (3 samples × 3 fields) were analyzed. Platelet density was calculated by normalizing platelet counts to the analyzed SEM image area (0.0142 mm^2^) and expressed as platelets·mm^–2^. Surface coverage (%) was estimated as the fraction of the image area occupied by adherent platelets. Values are reported as mean ± standard deviation (*n* = 9). Platelet morphology was classified as round (nonactivated), dendritic (partially activated), and spread (fully activated).

### 2.5 Antibacterial Studies

An antibacterial test on the samples was performed according to the ISO 22196 protocol for evaluating the antibacterial effect.^40^ Detailed information about the antibacterial test is published in our previous paper.^41^ Briefly, a bacterial culture suspension of *Escherichia coli* (*E. coli*) (10^5^ colony-forming units (CFU)/mL) was prepared, from which 0.1 mL was pipetted onto the sample surface. The samples were then incubated in the incubator (I-105 CK UV, Kambič) for 24 h at 37 °C in a humidity box. After incubation, *E. coli* on the surface was removed using 2.5 mL of sterilized PBS and 0.2 mL of this solution was taken for inoculation of *E. coli* in the agar plate at 37 °C for 24 h. The number of CFUs can then be determined. For convenient CFU counting, before inoculating *E. coli* into the agar plate, the initial solution was diluted further with PBS by a factor of 10 °-10^5^. The CFU/mL were calculated using an automated colony counter (Acolyte 3, Synbiosis).

### 2.6 Cell Culture

Human coronary artery endothelial cells (HCEC) and human coronary artery smooth muscle cells (HCASMC) were purchased from Lifeline Cell Technology (Frederick, MD, USA). HCEC and HCASMC were plated into 75 cm^2^ flasks (TPP, Trasadigen, Switzerland) at 37 °C in a humidified atmosphere at 5% CO_2_ and grown in VascuLife EnGS endothelial medium complete kit (Frederick, MD, USA) or VascuLife SMC medium (ProVitro AG, Berlin, Germany), respectively, following manufacturer’s instructions. For experiments, subconfluent cell cultures were used between passages 3 and 6.

### 2.7 Analysis

#### 2.7.1 Scanning Electron Microscopy (SEM and EDAX) Analysis

The morphological analysis of the samples was conducted by Scanning electron microscope (JEOL JSM-7600F and Zeiss CrossBeam550) at an accelerating voltage of 5–15 kV and beam currents of 100 pA to 1 nA. The test was done in triplicate, and only representative images are shown. EDX analysis was performed by an EDAX OctaneElite EDX detector, using Apex2 software.

#### 2.7.2 Water Contact Angle (WCA) Analysis

The surface wettability was performed with the Drop Shape Analyzer DSA-100 (Krüss GmbH, Hannover, Germany) by a sessile drop method to measure a static contact angle. The contact angle on the surface was analyzed immediately after plasma treatment or after storage in air at a constant temperature (21 °C) and humidity (45%). A drop of 2.5 μL of deionized water was placed on 8 different areas of the surface. Three measurements were performed for each sample, and the average value was calculated. The relative humidity was approximately 45%, and the operating temperature was 21 °C, which remained relatively constant during continuous measurements.

#### 2.7.3 X-ray Photoelectron Spectroscopy (XPS)

The X-ray photoelectron spectroscopy (XPS) analyses were carried out on the PHI-TFA XPS spectrometer produced by Physical Electronics Inc. Samples were put on the sample holder and were introduced into the ultrahigh vacuum spectrometer. The analyzed area was 0.4 mm in diameter, and the analyzed depth was approximately 3–5 nm. Sample surfaces were excited by X-ray radiation from a monochromatic Al source at a photon energy of 1486.6 eV.

#### 2.7.4 Immunofluorescent Microscopy and Cell Morphology

The HCEC and HCASMC were seeded on round disks of sample materials in 12-well plates at a density of 20,000 cells per cm^2^ and grown for 2 days. The test was conducted in biological duplicates. Fluorescein Phalloidin (Invitrogen) staining was performed according to the manufacturer’s instructions. Briefly, cells were washed twice for 3 min with PBS (pH 7.4), fixed in a 3.7% formaldehyde solution for 10 min, and washed three times for 3 min with PBS at room temperature. Cells were incubated in 0.1% Triton X-100 for 4 min, then washed three times with PBS for 3 min each. Dye stock was diluted 1:67 in PBS with 1% BSA and applied to HCEC and HCASMC for 30 min. Final washing steps were performed 3 times for 3 min with PBS. ProLong Diamond Antifade Mountant with DAPI (Thermo Fisher Scientific, USA) was applied to HCEC and HCASMC (1 drop) and covered with a coverslip. Slides were examined the same day. Images were generated using a Nikon Eclipse E 400 fluorescent microscope and a digital camera (Nikon Instruments, Düsseldorf, Germany). Analysis was performed with Nikon ACT-1 imaging software; the representative images are presented. Cell adhesion was quantified from fluorescence microscopy images acquired at identical magnification. For each surface, three independent fields were analyzed (*n* = 3), and adherent cells were manually counted and expressed as cells per field. HCEC and HCASMC were evaluated separately. Data are presented as mean ± standard deviation. The HCEC/HCASMC ratio was calculated from mean values to assess relative cell selectivity.

## 3 Results

### 3.1 Morphology

SEM analysis revealed that plasma-induced electrochemical anodization (PA) in deionized water (dH_2_O) resulted in sparsely distributed microstructures on the titanium surface (Figure 2a). Similar flower-like microstructures were observed when PA was performed in the other electrolytes; however, their surface coverage and uniformity varied significantly with electrolyte composition (Figure 2b–d). In the case of dH_2_O, the formation of surface features may be attributed to the generation of hydroxyl (–OH) radicals during water dissociation, leading to limited surface modification. The low ionic strength of dH_2_O, however, was insufficient to support uniform nanostructure formation, as evidenced by the sparse distribution of flower-like features. When phosphate-buffered saline (PBS) was used as the electrolyte, surface cracking accompanied by a low density of microstructures was observed (Figure 2b), which can be attributed to the relatively low molarity of chloride salts (KCl and NaCl) present in PBS. Increasing the chloride ion concentration by employing NaCl electrolytes resulted in a markedly more uniform distribution of flower-like microstructures on the surface (Figure 2c), indicating that chloride concentration plays a key role in initiating and sustaining nanostructure growth during PA. The influence of ethylene glycol (EG) content in NaCl-based electrolytes was further investigated by varying the EG/dH_2_O ratio. Increasing the EG fraction from 1:5 to 3:2 resulted in a significant reduction in the number of flower-like structures on the surface. The most homogeneous and well-defined morphology was obtained using an EG/dH_2_O ratio of 1:5 in the NaCl electrolyte (Figure 2d). These observations are consistent with previous reports demonstrating that water content critically influences nanotube formation during anodization.^5,42^

The influence of anodization parameters, specifically electrolyte concentration and treatment time, on surface morphology was further investigated (Figure 3). Increasing the chloride ion concentration had a pronounced effect on the formation of flower-like microstructures. A 2-fold increase in NaCl molarity resulted in a substantially more uniform and densely distributed morphology, with well-defined microflowers clearly observed by SEM (Figure 3c,d). In contrast, extending the anodization time from 30 to 60 min did not significantly affect flower formation, indicating that electrolyte concentration is the dominant factor governing nanostructure development under the applied conditions. Thus, the SEM results demonstrate that chloride ion concentration primarily controls both the quantity and uniformity of the flower-like structures formed during PA.

A more detailed analysis of the nanostructures produced by PA in 2.5 M NaCl with ethylene glycol for 60 min is presented in Figure 4. High-magnification SEM images (Figure 4b,d) reveal that the flower-like structures consist of bundles of closely packed nanotubes forming a hierarchical architecture. Their formation is attributed to localized chloride-induced attack and etching of the titanium surface, leading to nanotube nucleation at pit sites and subsequent growth into disordered bundles that protrude from the surface, resulting in the characteristic flower-like morphology (Figure 4b–d). Broken structures observed at the base of these features (Figure 4c) further support a breakdown-driven growth mechanism. The size distribution of the flower-like structures was evaluated from SEM micrographs (Figure 4). The structures exhibit lateral dimensions predominantly in the range of approximately 0.6–2.5 μm, with most features falling between 1.0 and 1.8 μm, and occasional larger structures up to ∼3 μm. This relatively broad size distribution reflects a heterogeneous nucleation-and-growth process governed by the stochastic nature of localized breakdown during plasma-induced anodization. Based on high-magnification SEM observations, the characteristic diameter of the elongated nanotubes is estimated to be on the order of approximately 50 nm. However, due to limitations in image resolution and contrast, the boundaries of individual nanotubes are not sufficiently well-defined to permit a reliable statistical evaluation of diameter distribution. Therefore, this value should be considered an approximate estimate derived from SEM observations. Such behavior is consistent with previous reports showing that chloride-containing electrolytes can induce passivity breakdown and pitting corrosion on titanium surfaces.^43,44^ Under these conditions, localized surface activation leads to high current densities at specific sites, promoting rapid nanotube formation. Variations in anodization parameters primarily influence the nucleation density and growth rate of these features rather than the geometry of individual structures. Consequently, the overall surface coverage and density of nanotubular bundles are strongly governed by the applied anodization voltage, with higher voltages generating more initiation sites and accelerating nanostructure formation.

**Figure 4:**
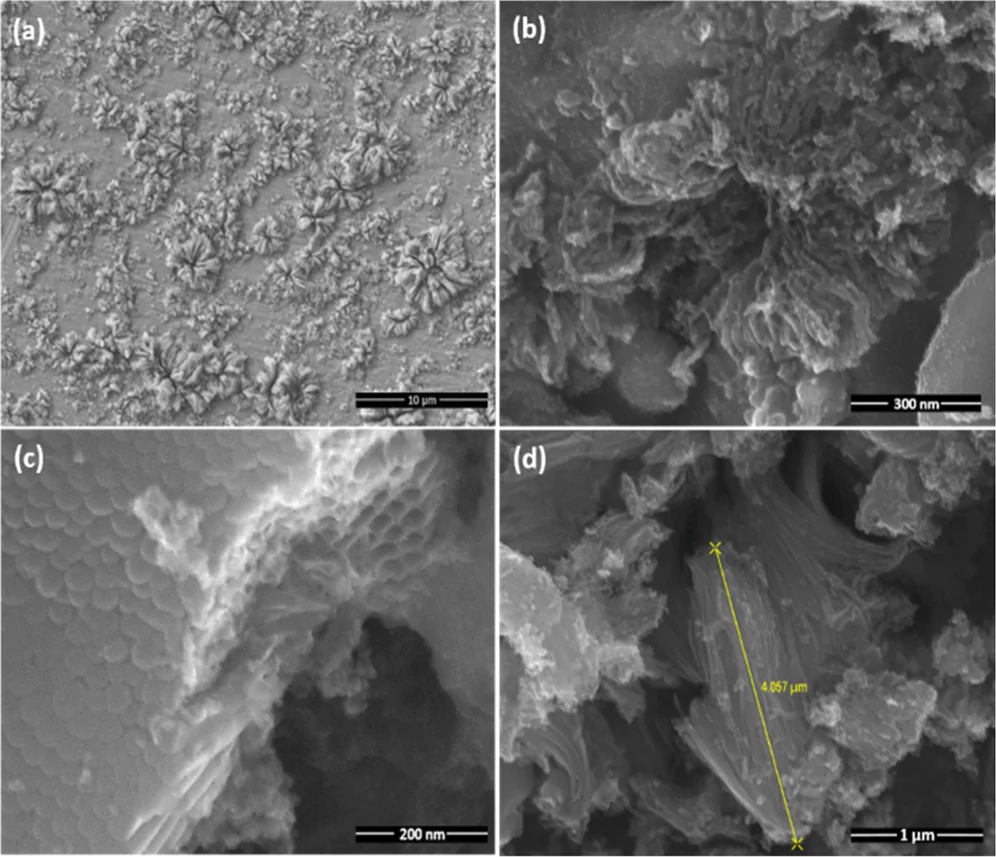
High-magnification SEM images of Ti foil subjected to plasma anodization in an electrolyte solution containing 2.5 M NaCl and ethylene glycol (EG) for 60 min, showing hierarchical flower-like TiO_2_ nanotubular structures at different magnifications: (a) overview morphology, (b,c) nanotubular bundle structures, and (d) detailed view of the nanotube-like architecture.

### 3.2 Elemental Composition

The surface chemical composition of the nanotubular flower structures and untreated titanium was analyzed by X-ray photoelectron spectroscopy (XPS), as summarized in Table 1. This analysis was performed to characterize the chemical composition of the PA-generated nanostructures and to assess the effects of subsequent gaseous plasma treatment. The untreated titanium surface consisted of approximately 43.6 at % oxygen, 39.3 at % carbon, and 17.1 at % titanium, corresponding to an O/Ti atomic ratio of 2.55.

**Table 1:** Surface Chemical Composition in Atomic % (at %) Obtained by XPS Analysis of Samples^a^

sample	atomic %				ratio	
	O	C	Ti	Cl	O/Ti	O/C
Ti	43.6	39.3	17.1	/	2.55	1.11
Ti + P	68.1	20.8	11.1	/	6.14	3.27
Ti + PA	45.0	36.8	16.7	1.5	2.69	1.22
Ti + PA + P	51.9	27.2	19.4	1.4	2.66	1.91

a The table includes O/Ti and O/C ratio.

Following oxygen plasma treatment (Ti + P), a pronounced increase in surface oxygen content and a corresponding reduction in carbon were observed, consistent with oxidative removal of adventitious carbon and the formation of a more stoichiometric, hydroxyl-rich TiO_2_ surface. In comparison, PA-treated samples (Ti + PA) exhibited a modest increase in oxygen relative to untreated titanium, together with a small but detectable chlorine content (∼1.5 at. %) originating from the NaCl-based electrolyte. For comparison, conventional electrochemical anodization with fluoride-containing electrolytes typically yields more than 2 at % residual fluoride on the surface.^42,45^ Energy-dispersive X-ray (EDX) analysis was performed to further verify the chemical composition of the fabricated TiO_2_ nanotubular flower structures (Figure 5). Elemental analysis was conducted at two distinct regions: areas containing TiO_2_ nanotubular flowers (Area 1) and regions of the anodized surface without flower-like structures (Area 2). The atomic percentages of the detected elements for both regions are summarized in the inset of Figure 5.

**Figure 5:**
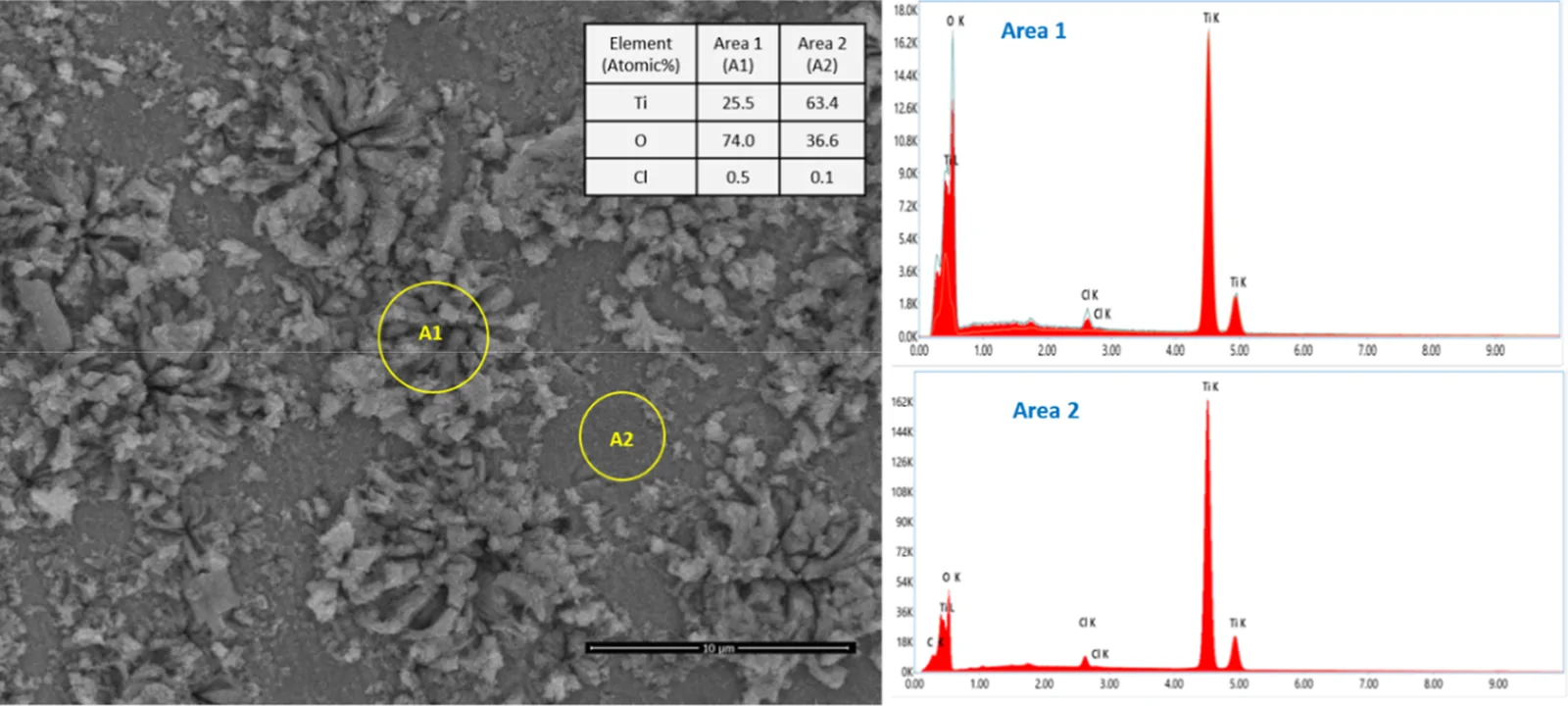
Energy dispersive X-ray analysis (EDAX) of two demarcated areas in SEM image of PA sample. Table in the inset of figure shows the atomic percentage of the constituent elements present in the specific area.

EDX analysis revealed a pronounced compositional difference between regions containing nanotubular flower structures (Area 1) and regions without flower formation (Area 2). Area 1 exhibited a substantially higher oxygen content (74%) compared to Area 2 (36%), while the titanium content was correspondingly lower in Area 1 (25.5%) than in Area 2 (63.4%). These results support the hypothesis that flower formation initiates at localized sites on the anodized surface, where enhanced oxidation leads to preferential nanotube growth. Such localized initiation results in less controlled growth and inhomogeneous distributions across the substrate, affecting both surface coverage and nanotube length. The nanotubes subsequently cluster into closely packed bundles, which assemble into the characteristic flower-like nanostructures. To further modify surface chemistry and wettability, low-pressure plasma treatment was applied to Ti + P and Ti + PA + P samples. As observed in the compositional analysis, plasma treatment increased the surface concentrations of oxygen and titanium while reducing carbon content, leading to pronounced changes in surface wettability. Water contact angle (WCA) measurements confirmed that plasma treatment significantly enhanced surface wettability; however, this effect was not equally stable for all samples. After 1 week of aging in air, Ti + PA and Ti + PA + P surfaces remained fully wettable (contact angle <40°), and only a slight increase in contact angle was observed after 3 weeks. Even after 4 weeks of aging, the contact angle remained low, at approximately 12°. In contrast, untreated titanium exhibited hydrophobic behavior with a contact angle of approximately 98°. Although plasma-treated titanium (Ti + P) initially displayed superhydrophilic behavior, this state was short-lived, with the contact angle increasing to approximately 75° after 3 weeks, indicating hydrophobic recovery.

### 3.3 Platelet Adhesion

Platelet interactions with the sample surfaces were examined by SEM, and representative images are shown in Figure 6, while quantitative analysis is summarized in Table 2. Untreated titanium exhibited a high density of adherent platelets, including both individual platelets and aggregates that were predominantly fully spread across the surface (Figure 6a). These platelets displayed dendritic and spread morphologies with well-developed lamellipodia and occasional pseudopodia, indicating a highly activated state. This observation is consistent with the quantitative data, which show the highest platelet density, (6.20 ± 0.25) × 10^3^ mm^–2^, and surface coverage (31.2 ± 2.8%). Morphological analysis further confirmed that the majority of platelets were fully spread (70.4%), with smaller contributions from dendritic (19.5%) and round (10.1%) forms, characteristic of a highly thrombogenic surface. Following plasma treatment (Ti + P), platelet adhesion was markedly reduced (Figure 6b). Only a few platelets were observed, predominantly exhibiting a rounded morphology, suggesting minimal activation. This is supported by the quantitative results, which show a substantial decrease in platelet density (190 ± 42 mm^–2^) and surface coverage (2.1 ± 0.7%). No spread platelets were detected, and the adherent population consisted mainly of dendritic (61.1%) and round (38.9%) morphologies. Evaluation of plasma-anodized surfaces (Ti + PA) was partially limited by the complex nanostructured topography (Figure 6c); however, only a very low number of platelet-like features were identified. Occasional spherical features resembling echinocytes were observed, which are commonly associated with artifacts arising from the anticoagulant used during blood collection.^46^ Quantitative analysis indicates low platelet density (162 ± 42 mm^–2^) and surface coverage (1.8 ± 0.6%), with all identifiable platelets exhibiting dendritic morphology (100%), suggesting partial activation without stable spreading. Due to the nanostructured background, platelet identification on these surfaces is uncertain, and only clearly distinguishable platelet features were included in the analysis. For samples subjected to both plasma-induced anodization and subsequent oxygen plasma treatment (Ti + PA + P), no discernible platelets or echinocytes were observed within any of the analyzed SEM fields (Figure 6d). This is consistent with the quantitative results, which show zero platelet density (0 ± 0 mm^–2^) and zero surface coverage (0 ± 0%, *n* = 9), indicating near-complete suppression of platelet adhesion. Overall, both plasma treatment and plasma-induced anodization significantly reduced platelet adhesion and inhibited platelet activation compared with untreated titanium. The Ti + PA + P treatment exhibited the strongest antithrombogenic behavior, as evidenced by no detectable platelets within the analyzed fields.

**Figure 6:**
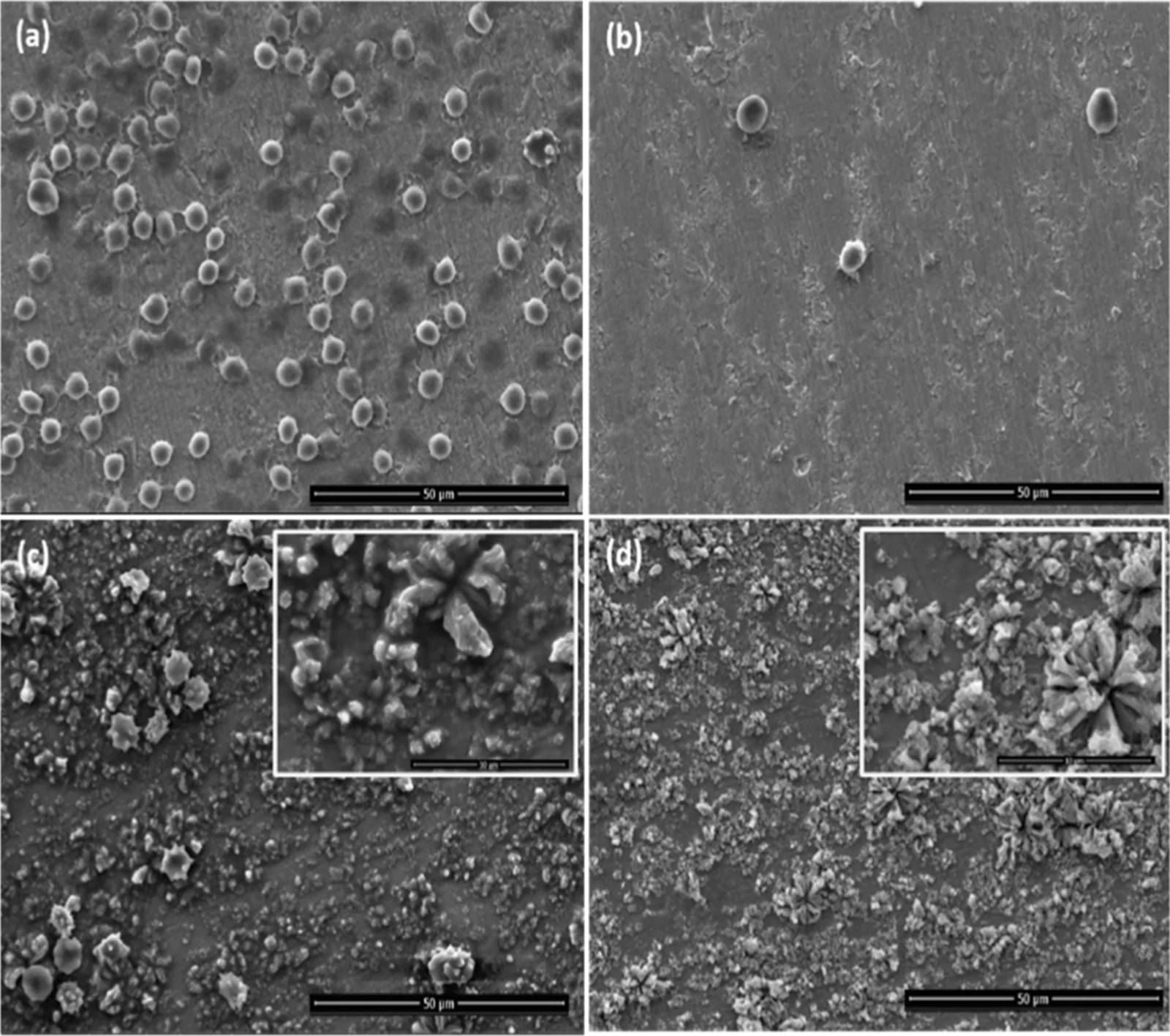
SEM images of (a) untreated Ti (b) Ti + P (c) Ti + PA and (d) Ti + PA + P after incubation with whole blood.

**Table 2:** Quantitative Analysis of Platelet Adhesion, Surface Coverage, and Morphology (*n* = 9 Fields per Sample)

sample	platelet density (mm^–2^, mean ± SD)	surface coverage (%)	round (cells/field (mean ± SD), % of total)	dendritic (cells/field (mean ± SD), % of total)	spread (cells/field (mean ± SD), % of total)
Ti	(6.20 ± 0.25) × 10^3^ mm^–2^	31.2 ± 2.8	8.9 ± 1.2 (10.1%)	17.2 ± 1.5 (19.5%)	61.9 ± 2.1 (70.4%)
Ti + P	190 ± 42	2.1 ± 0.7	1.0 ± 0.6 (38.9%)	1.7 ± 0.6 (61.1%)	0 ± 0 (0%)
Ti + PA	162 ± 42	1.8 ± 0.6	0 ± 0 (0%)	2.3 ± 0.6 (100%)	0 ± 0 (0%)
Ti + PA + P	0 ± 0	0 ± 0	not detectable	not detectable	not detectable

### 3.4 Antibacterial Studies

The antibacterial performance of TiO_2_ nanotubular flower surfaces was evaluated using *E. coli*, and the results are shown in Figure 7. Compared with untreated titanium, PA-treated samples (Ti + PA) exhibited approximately an 82% reduction in bacterial colony formation. Plasma-treated titanium (Ti + P) showed a moderate antibacterial effect, reducing *E. coli* colonies by 46% compared with untreated Ti. Notably, samples subjected to both plasma-induced anodization and subsequent oxygen plasma treatment (Ti + PA + P) showed the strongest antibacterial response, with an additional 55% reduction in bacterial attachment compared to Ti + PA (Figure 8). Overall, Ti + PA + P surfaces exhibited the lowest level of bacterial adhesion, with substantially fewer bacteria adhering to them. The reduced bacterial attachment can be attributed primarily to the nanoscale surface morphology of the TiO_2_ nanotubular flower structures. It has been widely reported that nanoscale surface features inhibit bacterial adhesion by limiting effective contact between bacterial cells and the substrate, thereby reducing surface-to-volume interactions. In the context of biomedical implants, final surface processing commonly includes sterilization procedures, which must not compromise surface topography or antibacterial functionality.^39^ Nanostructured surfaces are known to significantly influence bacterial adhesion and proliferation, as well as cellular behavior and platelet activation, underscoring the importance of maintaining surface integrity throughout processing.^41,47,48^

**Figure 7:**
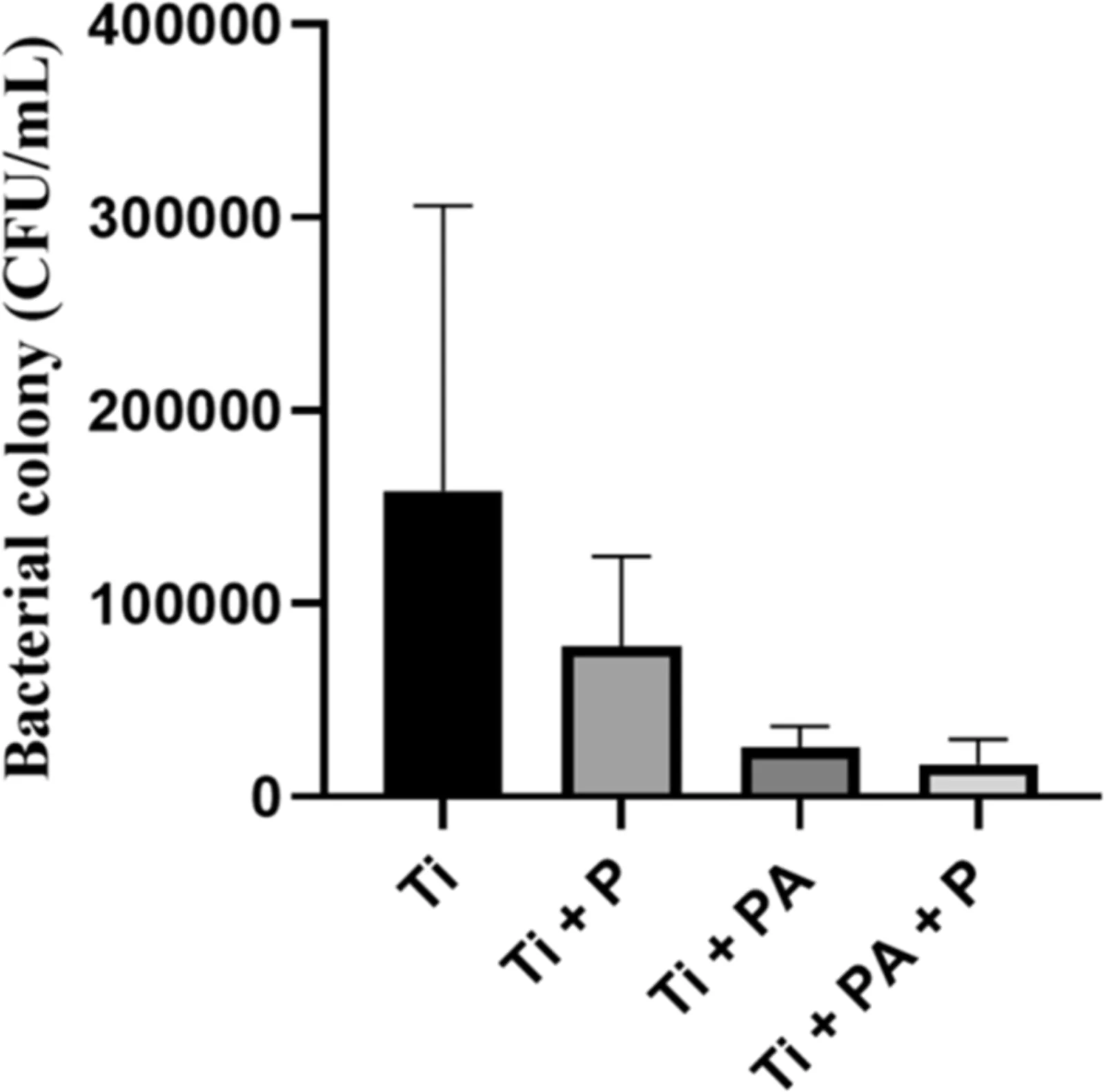
Adhesion of *E. coli* (mean CFU/mL) on TiO_2_ nanotubular (NT) flower surfaces: untreated Ti (Ti), Ti + P, Ti + PA, and Ti + PA + P. The figure shows the mean CFU/mL values obtained from four independent measurements for each surface.

**Figure 8:**
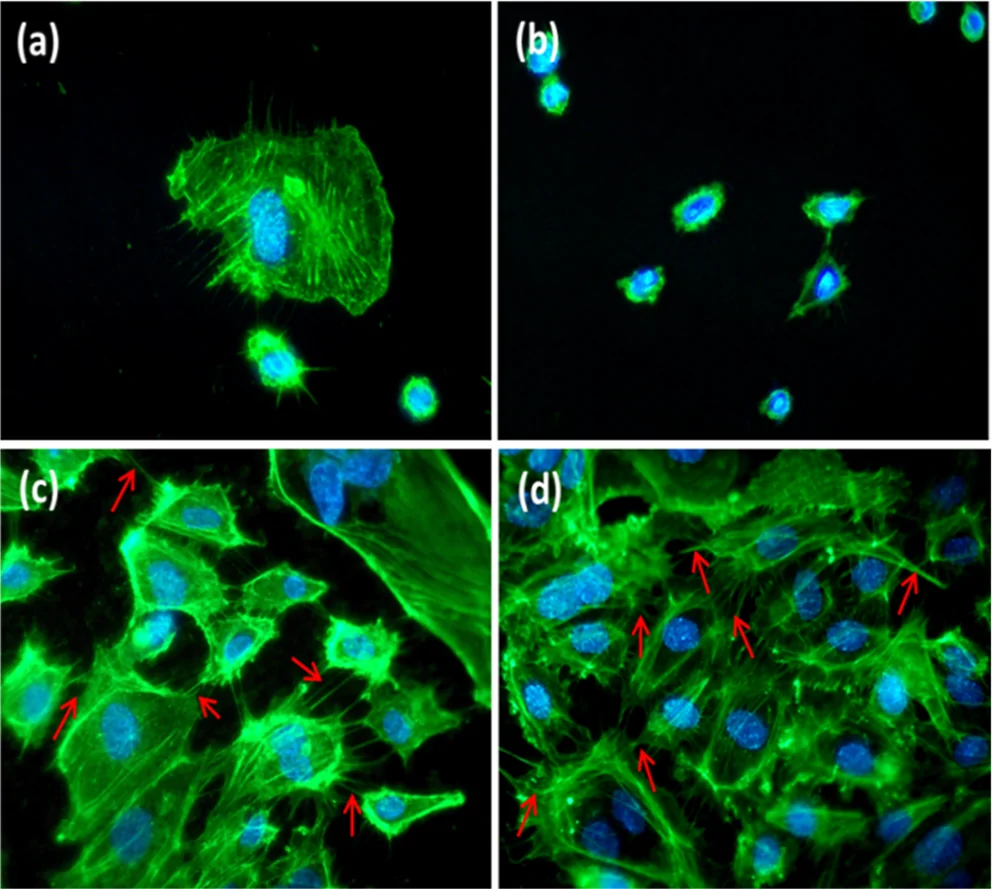
HCEC on the surface of (a) untreated Ti, (b) Ti + P, (c) Ti + PA, and (d) Ti + PA + P determined by immunofluorescent microscopy. F-actin is shown in green (FITC-Phalloidin). Nuclei are visualized with DAPI (blue color). Scale bar 25 μm. (Please denote by arrows long cytoplasmic extensions).

Bacterial attachment to nanostructured surfaces can be inhibited by reduced effective contact between bacterial cells and the substrate, thereby limiting stable adhesion.^49^ This effect arises from a mismatch between the characteristic dimensions of bacterial cells and the nanoscale surface features, resulting in insufficient contact area for firm attachment. In addition, bacteria may become physically trapped within surface trenches, pits, or recessed regions, further hindering stable colonisation.^50^ These mechanisms account for the antibacterial behavior observed for the fabricated TiO_2_ nanotubular flower surfaces, which also exhibit an oxygen-rich surface composition. The antibacterial effect is further enhanced in Ti + PA + P samples following oxygen plasma treatment, resulting in a 92% reduction in bacterial growth compared to untreated titanium.

### 3.5 Cell Studies

Using immunofluorescence microscopy, the morphology of human coronary artery endothelial cells (HCEC) on the sample surfaces was investigated. For vascular stent applications, rapid and stable endothelialisation is highly desirable, as a functional endothelial layer reduces thrombogenicity and improves hemocompatibility. Therefore, the interaction of endothelial cells with the proposed surface modifications was systematically examined.

As shown in Figure 8, the organization of actin filaments in HCEC (stained with FITC-phalloidin, green) revealed distinct cell morphologies across the investigated surfaces. On untreated titanium, HCEC exhibited limited spreading and reduced surface coverage, with cells maintaining a relatively rounded morphology. In contrast, plasma-treated and plasma-anodized surfaces promoted enhanced cell spreading, elongated morphology, and more pronounced cytoskeletal organization, together with more evident cell–cell interactions. On Ti + PA and Ti + PA + P surfaces, endothelial cells appeared flattened and extended long cytoplasmic processes (Figure 8c,d), indicating improved interaction with the substrate. Occasional membrane blebbing was observed on Ti + PA samples, whereas this feature was less pronounced after additional oxygen plasma treatment, suggesting a beneficial effect of surface chemistry modification. Notably, Ti + PA + P surfaces supported the most extensive and continuous endothelial cell spreading, consistent with improved surface compatibility and endothelialisation potential. Quantitative analysis supported these observations (Table 3), showing relatively low HCEC adhesion on untreated Ti surfaces (3.3 ± 0.6 cells per field), followed by a progressive increase after oxygen plasma treatment and plasma anodization (8.0 ± 1.0 and 16.7 ± 1.5 cells per field, respectively). The highest endothelial cell adhesion was observed on Ti + PA + P surfaces (22.3 ± 5.0 cells per field), indicating that the combination of plasma anodization and additional oxygen plasma treatment further enhanced endothelial cell attachment and spreading.

**Table 3:** Quantitative Analysis of Endothelial and Smooth Muscle Cell Adhesion on Unmodified and Modified Titanium Surfaces (Mean ± SD, *n* = 3)

sample	cell adhesion (cells per field, mean ± SD)		HCEC/HCASMC ratio
	HCEC	HCASMC	
Ti	3.3 ± 0.6	5.0 ± 1.0	0.7
Ti + P	8.0 ± 1.0	4.7 ± 1.5	1.7
Ti + PA	16.7 ± 1.5	21.7 ± 2.5	0.8
Ti + PA + P	22.3 ± 5.0	13.3 ± 1.5	1.7

The behavior of human coronary artery smooth muscle cells (HCASMC) is shown in Figure 9. On Ti and Ti + P surfaces, HCASMC exhibited relatively low cell density and less extensive spreading, although the cells retained a flattened morphology with visible cytoskeletal organization. In contrast, PA-treated surfaces (Ti + PA and Ti + PA + P) promoted markedly increased HCASMC adhesion and more elongated, nonuniform morphologies, with attachment occurring preferentially at specific regions of the nanostructured surface, suggesting modified cell–surface interactions induced by plasma anodization. Quantitative analysis confirmed relatively low HCASMC adhesion on Ti and Ti + P surfaces (5.0 ± 1.0 and 4.7 ± 1.5 cells per field, respectively), whereas plasma anodization substantially increased smooth muscle cell attachment, particularly on Ti + PA surfaces (21.7 ± 2.5 cells per field). Additional oxygen plasma treatment reduced HCASMC adhesion compared with Ti + PA, although values remained higher than on untreated surfaces (13.3 ± 1.5 cells per field).

**Figure 9:**
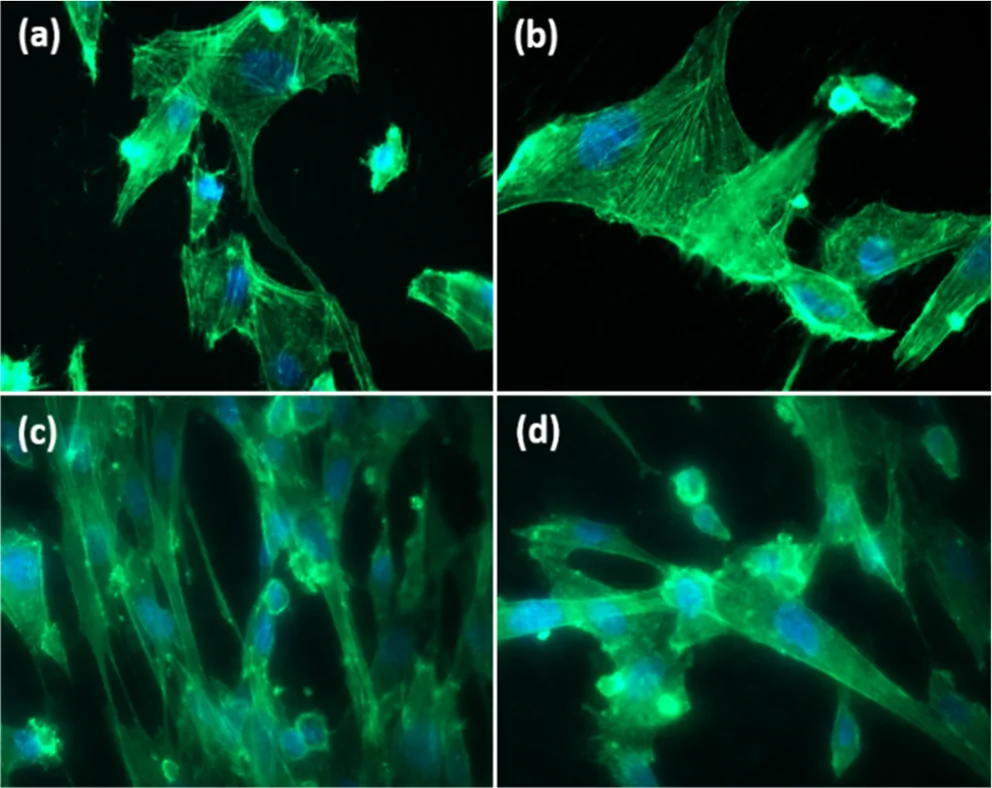
HCASMC on the surface of (a) untreated Ti (b) Ti + P (c) Ti + PA and (d) Ti + PA + P determined by immunofluorescent microscopy. F-actin is shown in green (FITC-Phalloidin). Nuclei are visualized with DAPI (blue). Scale bar 25 μm.

The ratio of HCEC to HCASMC adhesion provides additional insight into surface selectivity. Untreated Ti exhibited a ratio below unity (0.7), indicating preferential adhesion of smooth muscle cells. In contrast, Ti + P and Ti + PA + P surfaces exhibited the highest ratios (1.7), suggesting enhanced endothelial selectivity relative to smooth muscle cells. Although Ti + PA surfaces promoted strong adhesion of both cell types, the lower ratio (0.8) indicates less selective behavior. Overall, these results demonstrate that plasma-induced anodization strongly influences the adhesion behavior of both endothelial and smooth muscle cells. While Ti + PA surfaces promoted the highest HCASMC adhesion, the additional oxygen plasma treatment (Ti + PA + P) reduced smooth muscle cell attachment while maintaining enhanced endothelial adhesion, suggesting improved endothelial selectivity and promising behavior for cardiovascular implant applications.

## 4 Discussion

While plasma-assisted and fluoride-free anodization approaches have been reported, their scope and biological evaluation remain limited and fragmented. Conventional electrochemical anodization typically relies on fluoride-containing electrolytes to achieve self-organized TiO_2_ nanotube arrays with well-defined geometry.^51,52^ In contrast, fluoride-free systems based on chloride or other electrolytes generally yield less-ordered nanostructures due to the absence of controlled dissolution mechanisms. Under such conditions, morphology is governed by localized breakdown processes, resulting in irregular, disordered, or bundle-like features rather than uniform nanotube arrays. In this study, plasma-induced electrochemical anodization (PA) employing an atmospheric-pressure plasma jet as the cathode enables anodization in simple chloride-based electrolytes without the use of metallic counter-electrodes or hazardous fluoride species. This configuration promotes a chloride-induced localized breakdown mechanism, leading to the formation of hierarchical flower-like structures composed of nanotube-like features. These structures differ fundamentally from both the ordered arrays obtained in fluoride-based anodization and the less-defined morphologies typically reported in conventional fluoride-free systems. The morphology of the obtained surfaces strongly depended on electrolyte composition. Anodisation in deionized water resulted in only sporadic formation of flower-like structures, indicating insufficient ionic strength to sustain uniform breakdown and growth. Similarly, PBS led to nonuniform surface modification accompanied by cracking, likely due to its relatively low chloride content. In contrast, NaCl-based electrolytes, particularly at higher molarity, enabled more extensive and uniform formation of nanoflower structures, confirming the critical role of chloride ions in initiating and sustaining localized anodic breakdown. The addition of ethylene glycol further influenced nanostructure formation, suggesting that electrolyte viscosity and ion mobility regulate growth kinetics and structural organization. High-magnification SEM analysis revealed that the flower-like structures consist of bundles of closely packed nanotube-like features with variable orientations. This morphology reflects a stochastic nucleation-and-growth process, in which localized breakdown sites serve as initiation points for structure formation. The resulting size distribution (∼0.6–3 μm) further supports the heterogeneous nature of the process. The characteristic diameter of the nanotube-like features was estimated to be approximately 50 nm based on SEM observations, although a precise statistical evaluation was not possible due to resolution limitations. Surface chemical analysis confirmed that the nanostructured layers are primarily composed of TiO_2_, with only minor chlorine incorporation from the electrolyte. Oxygen plasma treatment further increased oxygen content and reduced surface carbon contamination without altering morphology. This modification had a pronounced effect on wettability. While plasma-treated flat titanium exhibited hydrophobic recovery over time, PA-treated surfaces retained low contact angles even after prolonged aging, indicating that surface topography plays a key role in stabilizing wettability. The stable hydrophilic behavior of PA-treated surfaces can be attributed to their hierarchical architecture, combining nanoscale (∼50 nm) and microscale (∼0.6–3 μm) features. This multiscale roughness increases the effective surface area and promotes capillary-driven wetting, resulting in persistent hydrophilicity. In contrast, the wettability of Ti + P is governed primarily by surface chemistry and is therefore more susceptible to aging-induced hydrophobic recovery. The hierarchical morphology also plays a critical role in governing biological interactions. At the nanoscale, the dimensions of surface features are significantly smaller than those of bacterial cells, limiting effective contact and reducing stable adhesion. At the microscale, the flower-like structures provide topographical cues that influence mammalian cell behavior, including adhesion and spreading. The combined presence of multiple length scales further disrupts uniform bacterial colonisation while supporting endothelial cell organization. These effects are consistent with the experimental observations. A substantial reduction in bacterial adhesion was observed on PA-treated surfaces, particularly after additional oxygen plasma treatment. Similarly, platelet adhesion and activation were strongly suppressed, with no detectable platelets observed within the analyzed fields for Ti + PA + P samples. This indicates that the combined influence of surface topography and chemistry effectively interferes with early stage platelet–surface interactions. Cell studies further demonstrate the biological selectivity of the PA-treated surfaces. Endothelial cells exhibited enhanced adhesion, spreading, and cytoskeletal organization, particularly on Ti + PA + P surfaces, indicating favorable conditions for endothelialisation. In contrast, smooth muscle cells showed altered morphologyand nonuniform distribution on PA-treated surfaces, suggesting modified interactions with the nanostructured surface. Although adhesion of both cell types increased on modified surfaces, the more pronounced response of endothelial cells indicates a beneficial trend toward selective endothelialisation. Overall, the results demonstrate that plasma-induced electrochemical anodization provides an effective strategy for engineering hierarchical nanostructures on titanium surfaces and tailoring surface chemistry. The resulting surfaces exhibit reduced platelet adhesion, diminished bacterial attachment, and a favorable cellular response characterized by enhanced endothelial behavior and moderated smooth muscle cell interaction. Importantly, these effects are achieved using environmentally benign electrolytes, highlighting the potential of the PA approach for the development of advanced blood-contacting biomedical devices.

## 5 Conclusions

This study demonstrates that plasma-induced electrochemical anodization (PA) enables the formation of titanium oxide nanostructures via a fundamentally different growth mechanism from the self-organized nanotubular arrays typically obtained in fluoride-containing electrolytes. In the PA process, using an atmospheric-pressure plasma jet as the cathode enables anodization in environmentally benign, chloride-based electrolytes, thereby eliminating the need for strong acids and metallic counter-electrodes. The resulting nanostructures arise from localized passivity breakdown, leading to the formation of hierarchical, flower-like microstructures composed of densely packed TiO_2_ nanotube bundles. Systematic variation of anodization parameters revealed that chloride ion concentration is the primary factor governing nanostructure density and surface coverage, while electrolyte composition and water content influence nanotube length and morphological definition. Among the investigated electrolytes, NaCl with ethylene glycol yielded the most uniform and well-defined nanotubular flower morphologies. XPS and EDX analyses confirmed that the structures consist predominantly of TiO_2_, with only minor chlorine incorporation from the electrolyte. Subsequent low-pressure oxygen plasma treatment modified surface chemistry by increasing surface oxygen content and reducing carbon contamination, without altering surface topography, resulting in enhanced, more stable wettability. The physicochemical properties of the PA-treated surfaces translated into distinct biological responses. Platelet adhesion and activation were strongly suppressed on nanotubular flower surfaces, indicating reduced thrombogenic potential. Bacterial attachment of *E. coli* was significantly reduced, with the strongest effect observed after additional oxygen plasma treatment. Cell studies further demonstrated a selective response, with enhanced spreading and cytoskeletal organization of endothelial cells, while smooth muscle cells exhibited altered morphology and modified cell–surface interactions on PA-treated surfaces. Taken together, these findings indicate that plasma-induced electrochemical anodization provides a robust, environmentally sustainable route for tailoring the morphology and chemistry of titanium surfaces. The combination of a hierarchical nanotubular architecture and a favorable biological response highlights the potential of this approach for developing titanium-based surfaces for cardiovascular stents and other blood-contacting biomedical devices.

## References

[1] Lee K., Mazare A., Schmuki P. (2014). One-dimensional titanium dioxide
nanomaterials: nanotubes. Chem. Rev..

[2] Bjursten L. M., Rasmusson L., Oh S., Smith G. C., Brammer K. S., Jin S. (2010). Titanium dioxide nanotubes enhance bone bonding in vivo. J. Biomed. Mater. Res., Part A.

[3] Nian J.-N., Teng H. (2006). Hydrothermal synthesis of single-crystalline anatase TiO_2_ nanorods with nanotubes as the precursor. J. Phys. Chem. B.

[4] Kulkarni M., Mazare A., Gongadze E., Perutkova S. ˇ., Kralj-Iglič V., Milošev I., Schmuki P., Iglič A., Mozetič M. (2015). Titanium nanostructures
for biomedical applications. Nanotechnology.

[5] Cheong Y. L., Yam F. K., Ng S. W., Hassan Z., Ng S. S., Low I. M. (2015). Fabrication of titanium dioxide nanotubes
in fluoride-free
electrolyte via rapid breakdown anodization. J. Porous Mater..

[6] Babelon P., Dequiedt A. S., Mostefa-Sba H., Bourgeois S., Sibillot P., Sacilotti M. (1998). SEM and XPS
studies of titanium dioxide
thin films grown by MOCVD. Thin Solid Films.

[7] Macak J. M., Tsuchiya H., Taveira L., Aldabergerova S., Schmuki P. (2005). Smooth anodic TiO_2_ nanotubes. Angew. Chem., Int. Ed..

[8] Kulkarni M., Mazare A., Park J., Gongadze E., Killian M. S., Kralj S., von der Mark K., Iglič A., Schmuki P. (2016). Protein interactions with layers of TiO_2_ nanotube and nanopore arrays: Morphology and surface charge influence. Acta Biomater..

[9] Wang D., Liu Y., Yu B., Zhou F., Liu W. (2009). TiO_2_ Nanotubes
with Tunable Morphology, Diameter, and Length: Synthesis and Photo-Electrical/Catalytic
Performance. Chem. Mater..

[10] Benčina M., Iglič A., Mozetič M., Junkar I. (2020). Crystallized TiO_2_ nanosurfaces in biomedical applications. Nanomaterials.

[11] Macak J. M., Tsuchiya H., Ghicov A., Yasuda K., Hahn R., Bauer S., Schmuki P. (2007). TiO_2_ nanotubes: Self-organized
electrochemical formation, properties and applications. Curr. Opin. Solid State Mater. Sci..

[12] Ishibashi K.-i., Yamaguchi R.-t., Kimura Y., Niwano M. (2008). Fabrication
of Titanium
Oxide Nanotubes by Rapid and Homogeneous Anodization in a Mixture
of Perchloric Acid and Ethanol. J. Electrochem.
Soc..

[13] Liu Y., Mu K., Zhang Y., Wang L., Yang G., Shen F., Deng S., Zhang X., Zhang S. (2016). Facile synthesis of
a narrow-gap titanium dioxide anatase/rutile nanofiber film on titanium
foil with high photocatalytic activity under sunlight. Int. J. Hydrogen Energy.

[14] Liu Y., Mu K., Zhong J., Chen K., Zhang Y., Yang G., Wang L., Deng S., Shen F., Zhang X. (2015). Preparation
of a sunlight-driven TiO_2_ film with excellent material
characterizations and photoelectrocatalytic properties by an eco-friendly
manner. RSC Adv..

[15] Sreekantan S., Wei L. C., Lockman Z. (2011). Extremely fast growth rate of TiO_2_ nanotube arrays in electrochemical bath containing H_2_O_2_. J. Electrochem. Soc..

[16] Hahn R., Lee H., Kim D., Narayanan S., Berger S., Schmuki P. (2008). Self-organized
anodic TiO_2_-nanotubes in fluoride free electrolytes. ECS Trans..

[17] Richter C., Panaitescu E., Willey R., Menon L. (2007). Titania nanotubes prepared
by anodization in fluorine-free acids. J. Mater.
Res..

[18] Huang X., Li Y., Zhong X. (2014). Temperature-induced
reversible self-assembly of diphenylalanine
peptide and the structural transition from organogel to crystalline
nanowires. Nanoscale Res. Lett..

[19] Höfft O., Endres F. (2011). Plasma electrochemistry in ionic liquids: an alternative
route to generate nanoparticles. Phys. Chem.
Chem. Phys..

[20] Brettholle M., Hoefft O., Klarhöfer L., Mathes S., Maus-Friedrichs W., El Abedin S. Z., Krischok S., Janek J., Endres F. (2010). Title not
provided please verify. Phys. Chem. Chem. Phys..

[21] Hieda J., Saito N., Takai O. (2008). Exotic shapes
of gold nanoparticles
synthesized using plasma in aqueous solution. J. Vac. Sci. Technol., A.

[22] Omurzak E., Jasnakunov J., Mairykova N., Abdykerimova A., Maatkasymova A., Sulaimankulova S., Matsuda M., Nishida M., Ihara H., Mashimo T. (2007). Synthesis method of nanomaterials
by pulsed plasma in liquid. J. Nanosci. Nanotechnol..

[23] Ambujakshan A. T., Sadek A., Magniez K., Mateti S., Mayes E., Devi G., Pringle J. M., Plessis J. d., Chen Z., Corr C. S. (2017). Plasma treated water–A
promising electrolyte
to produce nanoporous titanium dioxide nanotubes. Plasma Processes Polym..

[24] Dai X. J., Corr C. S., Ponraj S. B., Maniruzzaman M., Ambujakshan A. T., Chen Z., Kviz L., Lovett R., Rajmohan G. D., De Celis D. R. (2016). Efficient
and selectable
production of reactive species using a nanosecond pulsed discharge
in gas bubbles in liquid. Plasma Processes Polym..

[25] Allam N. K., Shankar K., Grimes C. A. (2008). Photoelectrochemical
and water photoelectrolysis
properties of ordered TiO_2_ nanotubes fabricated by Ti anodization
in fluoride-free HCl electrolytes. J. Mater.
Chem..

[26] Lin C.-J., Yu W.-Y., Chien S.-H. (2008). Rough conical-shaped
TiO_2_-nanotube arrays for flexible backilluminated dye-sensitized
solar
cells. Appl. Phys. Lett..

[27] Ambujakshan A. T., Pringle J. M., Chen Z., Corr C. S., du Plessis J., Hodgson P. D., Dai X. J. (2018). An environmentally friendly in situ
plasma and anodization method to produce titanium dioxide nanotubes. Plasma Processes Polym..

[28] Mariotti D., Sankaran R. M. (2011). Perspectives on
atmospheric-pressure plasmas for nanofabrication. J. Phys. D: Appl. Phys..

[29] Ostrikov K., Neyts E. C., Meyyappan M. (2013). Plasma nanoscience:
from nano-solids
in plasmas to nano-plasmas in solids. Adv. Phys..

[30] Laroussi M., Lu X. (2005). Room-temperature atmospheric
pressure plasma plume for biomedical
applications. Appl. Phys. Lett..

[31] Pai D. Z., Kumar S., Levchenko I., Lacoste D. A., Laux C. O., Ostrikov K. (2011). Atmospheric-pressure discharges for the fabrication
of surface-based metal nanostructures. IEEE
Trans. Plasma Sci..

[32] Fang J., Levchenko I., Ostrikov K. K. (2015). Atmospheric plasma jet-enhanced anodization
and nanoparticle synthesis. IEEE Trans. Plasma
Sci..

[33] Kuo Y.-L., Kencana S. D., Lin Y.-J. (2021). Atmospheric
pressure plasma jet fabricating
of porous silver electrocatalyst as a promising approach to the creation
of cathode layers of low temperature solid oxide fuel cells. Surf. Coat. Technol..

[34] Levchenko I., Ostrikov K. K., Zheng J., Li X., Keidar M., B K Teo K. (2016). Scalable graphene production: Perspectives
and challenges
of plasma applications. Nanoscale.

[35] Bazaka K., Baranov O., Cvelbar U., Podgornik B., Wang Y., Huang S., Xu L., Lim J. W. M., Levchenko I., Xu S. (2018). Oxygen plasmas: a sharp chisel and
handy trowel for nanofabrication. Nanoscale.

[36] Jadwiszczak J., O’Callaghan C., Zhou Y., Fox D. S., Weitz E., Keane D., Cullen C. P., O’Reilly I., Downing C., Shmeliov A. (2018). Oxide-mediated recovery
of field-effect mobility in plasma-treated MoS_2_. Sci. Adv..

[37] Benčina M., Junkar I., Zaplotnik R., Valant M., Iglič A., Mozetič M. (2019). Plasma-Induced Crystallization of TiO_2_ Nanotubes. Materials.

[38] Junkar I., Cvelbar U., Vesel A., Hauptman N., Mozetič M. (2009). The role of
crystallinity on polymer interaction with oxygen plasma. Plasma Processes Polym..

[39] Junkar I., Kulkarni M., Drašler B., Rugelj N., Mazare A., Flašker A., Drobne D., Humpolíček P., Resnik M., Schmuki P. (2016). Influence of various
sterilization procedures on TiO_2_ nanotubes used for biomedical
devices. Bioelectrochemistry.

[40] Rawat N., Benčina M., Paul D., Kovač J., Lakota K., Žigon P., Kralj-Iglič V., Ho H. C., Vukomanović M., Iglič A. (2023). Fine-Tuning the Nanostructured Titanium Oxide
Surface for Selective
Biological Response. ACS Appl. Bio Mater..

[41] Rawat N., Benčina M., Gongadze E., Junkar I., Iglič A. (2022). Fabrication
of Antibacterial TiO_2_ Nanostructured Surfaces Using the
Hydrothermal Method. ACS Omega.

[42] Kulkarni M., Mrak-Poljsak K., Flašker A. (2015). Fabrication of TiO_2_ nanotubes
for bioapplications. Mater. Tehnol..

[43] Szklarska-Smialowska Z. (1986). Pitting Corrosion
of Metals: A Review of the Critical Factors, Mechanisms and Testing
Methods. Corrosion.

[44] Hahn R., Macak J. M., Schmuki P. (2007). Rapid anodic growth
of TiO_2_ and WO_3_ nanotubes in fluoride free electrolytes. Electrochem. Commun..

[45] Junkar, I. ; Kulkarni, M. ; Humpolíček, P. ; Capáková, Z. ; Burja, B. ; Mazare, A. ; Schmuki, P. ; Mrak-Poljšak, K. ; Flašker, A. ; Žigon, P. ; Junkar, I. , Could titanium dioxide nanotubes represent a viable support system for appropriate cells in vascular implants? In Advances in Biomembranes and Lipid Self-Assembly; Elsevier: 2017.

[46] Walker, H. K. ; Hall, W. D. ; Hurst, J. W. Clinical Methods: The History, Physical, and Laboratory Examinations, 3rd ed.; Butterworths: Boston, 1990. 21250045

[47] Narendrakumar K., Kulkarni M., Addison O., Mazare A., Junkar I., Schmuki P., Sammons R., Iglič A. (2015). Adherence
of oral streptococci to nanostructured titanium surfaces. Dent. Mater..

[48] Llopis-Grimalt M. A., Forteza-Genestra M. A., Alcolea-Rodriguez V., Ramis J. M., Monjo M. (2020). Nanostructured
titanium for improved endothelial biocompatibility and reduced platelet
adhesion in stent applications. Coatings.

[49] Thomas V., Dean D. R., Vohra Y. K. (2006). Nanostructured biomaterials
for regenerative
medicine. Curr. Nanosci..

[50] Kargar M., Wang J., Nain A. S., Behkam B. (2012). Controlling
bacterial
adhesion to surfaces using topographical cues: a study of the interaction
of Pseudomonas aeruginosa with nanofiber-textured surfaces. Soft Matter.

[51] Regonini D., Bowen C. R., Jaroenworaluck A., Stevens R. (2013). A Review of Growth
Mechanism, Structure and Crystallinity of Anodized TiO_2_ Nanotubes. Mater. Sci. Eng. R Rep..

[52] Macak J. M., Schmuki P. (2006). Anodic Growth of Self-Organized
TiO_2_ Nanotubes
in Viscous Electrolytes. Electrochim. Acta.

